# Determinants of operative time in thyroid surgery: A prospective multicenter study of 3454 thyroidectomies

**DOI:** 10.1371/journal.pone.0181424

**Published:** 2017-07-27

**Authors:** Arnaud Patoir, Cécile Payet, Jean-Louis Peix, Cyrille Colin, Léa Pascal, Jean-Louis Kraimps, Fabrice Menegaux, François Pattou, Frédéric Sebag, Sandrine Touzet, Stéphanie Bourdy, Jean-Christophe Lifante, Antoine Duclos, on behalf of the CATHY Study Group

**Affiliations:** 1 Hospices Civils de Lyon, Centre Hospitalier Lyon Sud, Service de Chirurgie Générale et Endocrinienne, Pierre Bénite, France; 2 Hospices Civils de Lyon, Pôle Information Médicale Evaluation Recherche, Lyon, France; 3 Health Services and Performance Research Lab (EA 7425 HESPER), Université Claude Bernard Lyon 1, Lyon, France; 4 Department of Endocrine Surgery, Poitiers University, Jean Bernard Hospital, Poitiers, France; 5 Assistance Publique - Hôpitaux de Paris, Hôpital la Pitié-Salpêtrière, Service de Chirurgie Générale, Viscérale et Endocrinienne, Paris, France; 6 CHRU de Lille, Chirurgie Générale et Endocrinienne, Lille, France; Université Lille nord de France, INSERM, Lille, France; 7 Assistance Publique-Hôpitaux de Marseille, CHU la Timone-Adulte, France; 8 Center for Surgery and Public Health, Brigham and Women's Hospital, Harvard Medical School, Boston, Massachusetts, United States of America; Virginia Commonwealth University, UNITED STATES

## Abstract

**Objective:**

To identify the determinants of operative time for thyroidectomy and quantify the relative influence of preoperative and intra-operative factors.

**Background:**

Anticipation of operative time is key to avoid both waste of hospital resources and dissatisfaction of the surgical staff. Having an accurate and anticipated planning would allow a rationalized operating room use and may improve patient flow and staffing level.

**Methods:**

We conducted a prospective, cross-sectional study between April 2008 and December 2009. The operative time of 3454 patients who underwent thyroidectomy performed by 28 surgeons in five academic hospitals was monitored. We used multilevel linear regression to model determinants of operative time while accounting for the interplay of characteristics specific to surgeons, patients, and surgical procedures. The relative impact of each variable on operative time was estimated.

**Results:**

Overall, 86% (99% CI 83 to 89) of operative time variation was related to preoperative variables. Surgeon characteristics accounted for 32% (99% CI 29 to 35) of variation, center location for 29% (99% CI 25 to 33), and surgical procedure or patient variables for 24% (99% CI 20 to 27). Operative time was significantly lower among experienced surgeons having practiced from 5–19 years (-21.8 min, *P*<0.05), performing at least 300 thyroidectomies per year (-28.8 min, *P*<0.05), and with increasing number of thyroidectomies performed the same day (-11.7min, *P*<0.001). Conversely, operative time increased in cases of procedure supervision by a more experienced surgeon (+20.0 min, *P*<0.001). The remaining 13.0% of variability was attributable to unanticipated technical difficulties at the time of surgery.

**Conclusions:**

Variation in thyroidectomy duration is largely explained by preoperative factors, suggesting that it can be accurately anticipated. Prediction tools allowing better regulation of patient flow in operating rooms appears feasible for both working conditions and cost management.

## Introduction

In an era of cost-constrained health care, operating rooms are potential areas for cost reduction efforts. Hospital surgical suites can consume 9% of an institution’s annual budget; therefore it is necessary to schedule expensive surgical resources and use them efficiently [1]. In particular, anticipation of operative time is key to avoid both waste of resources and dissatisfaction of the surgical staff. Having an accurate and anticipated planning would allow a rationalized operating room use and may improve patient flow and staffing level [2]. Deviation from the scheduled time and the actual time required for a given procedure can disrupt the operating room program. When surgery takes longer than predicted, subsequent procedures may be postponed or cancelled, after which postoperative bed occupancy may be diminished [3]. When the duration of surgery is shorter than predicted, valuable time of surgical staffs is wasted and dissatisfaction may occur. Both situations also alter the working environment of surgeon and contributes to suboptimal use of operating room resources [4]. In effect, net staffing costs associated with different-than-average surgical durations are well quantified, and optimizing operative time prediction may reduce the costly overutilized and staffed hours [5].

For these reasons, adequate tools are needed to plan surgical procedures. The more accurately that preoperative parameters influencing operative time can be determined, the more reliable operating room scheduling will be. Thyroid surgery represents a good model to address this issue, as it traditionally consists of a limited range of highly standardized elective procedures [6]. According to the operative indication, thyroidectomy consists of a succession of well-described steps, which enables investigation into the influence of both preoperative and intraoperative factors on the procedure duration.

In this prospective multicenter study, we aimed to identify the determinants of operative time for thyroidectomy and quantify the relative influence of preoperative and intraoperative factors. For this purpose, we considered the interplay of several characteristics specific to surgeons, patients, and surgical procedures.

## Methods

### Study design and population

We conducted a prospective, cross-sectional study between April 1, 2008 and December 31, 2009 in five high-volume referral centers in France[7]. All 28 endocrine surgeons who performed thyroid surgery at these centers participated in the study. All patients who underwent a thyroid procedure were eligible for inclusion. The extent of inclusion was measured in relation to the number of eligible thyroidectomies recorded in the hospital administrative databases.

This study was approved by the Research Committee for the Protection of Persons (CPP) and the National Advisory Committee on Information Processing in Material Research in the Field of Health (CCTIRS) France, in accordance with French ethical directives. Informed consent was obtained from participating surgeons. The ethics committee waived the requirement for patient consent. Before surgery, patients received written information about personal data use and gave verbal consent for sharing their data.

### Outcome measures and data collection

The primary outcome was operative time, measured in minutes (min) and defined as the total duration from skin incision to closure of the wound. After each thyroidectomy, a patient report form was completed by the attending surgeon, including items about surgical indication and procedure, as well as the surgeon’s identity, the presence of a more experienced supervisory surgeon during the intervention, and the number of surgical procedures performed by that surgeon the same day. Research assistants completed data collection using medical records. These data included patient demographics, information on previous thyroid surgeries, and weight of the thyroid specimens.

Variables were divided into two groups: preoperative and intraoperative. Preoperative variables corresponded to information available before the operation. The center was characterized depending on the hospital where the procedure was performed. The surgeon’s length of experience was calculated as the number of years she/he had spent in practice since the completion of residency. Before the analysis, we separated the experience variable into four categories to reflect the successive steps in a surgeon’s career in France: less than 2 years (that is, a beginning surgeon starting a surgical fellowship), 2 to 4 years (junior surgeon ending a surgical fellowship), 5 to 19 years (senior surgeon), and 20 years and over (very experienced surgeon at the head of a surgical department). Additionally, the number of thyroidectomies performed by the surgeon on the same day was determined, as well as the volume of thyroidectomies that he/she had performed over the course of the year. Patient and procedure preoperative characteristics included sex, age, thyroid disease, body mass index, planned unilateral or bilateral thyroidectomy, lymph node dissection, and supervision by a more experienced surgeon. Intraoperative variables corresponded to technical challenges that occurred during the surgery, including difficulties in locating the parathyroid gland or in locating at least one recurrent nerve, large goiter, diving goiter, hemorrhagic goiter, fibrosis, thyroiditis, invasive cancer, and weight of the thyroid specimen.

### Statistical analysis

Data analyses were performed using SAS and R softwares. Operative time was described using means and standard deviation and compared using the Mann-Whitney test or Kruskal-Wallis test, according to the number of groups. All tests were two tailed, and *P* < 0.05 was considered statistically significant. To explore the determinants of operative time, we used a multilevel linear regression model in which patients were nested in surgeons, who in turn were nested in hospitals [8]. In this model, we classified center variables as a random effect, surgeon variables as both random and fixed effects, and all patient variables as fixed effects. A log transformation on operative time was performed to meet normality, which was successfully checked by using residuals and quantile-quantile plot. Results were presented in the original scale, as differences compared to the intercept with corresponding 95% confidence intervals (CI) calculated with the function deltamethod of the msm R-package [9]. To estimate the relative importance of variables to operative time, a multiple linear model was then performed including patients, surgeons, and center variables. The LMG metric via the R-package RELAIMPO was used to assess the relative importance of each variable and of groups of variables by decomposing the overall *R*^*2*^ into nonnegative *R*^*2*^ values for each variable and for each group of variables [10]. Relative importance estimates were then adjusted to sum to 100% for easier interpretation. Confidence intervals were computed via the same R-package with the percentile interval method of bootstrapping based on 1000 replicates, at a 99% confidence level to adjust for multiple comparisons and because these bootstrap CI can be somewhat liberal.

## Results

Of 3679 eligible thyroidectomies, 3454 (94%) were completed within the study period and selected for the analysis of operative time. Table 1 presents operative time according to characteristics of centers, surgeons, patients, and procedures.

**Table 1:** Operative time by population characteristics.

Population characteristics^a^		N = 3454 (%)	Operative time (min)	
			Mean	SD
***Preoperative variables***				
**Center**				
Localization	Hospital A	558 (16.1)	140.2	58.7
	Hospital B	739 (21.4)	60.7	31.3
	Hospital C	690 (20.0)	86.2	37.4
	Hospital D	973 (28.2)	102.3	44.6
	Hospital E	494 (14.3)	83.3	32.0
**Surgeon**				
Length of experience^c^ (years)	<2	333 (9.6)	138.3	52.6
	2–4	996 (28.8)	94.0	40.1
	5–19	760 (22.0)	97.5	42.7
	≥20	1365 (39.6)	80.2	49.8
No. of procedures performed on same day	1	233 (6.8)	123.4	63.2
	2	656 (19.0)	110.4	54.5
	3	1333 (38.6)	93.3	44.2
	≥4	1126 (32.6)	75.3	37.6
No. of thyroidectomies performed per year	<100	568 (16.4)	114.7	53.4
	100–199	1123 (32.5)	113.9	51.7
	200–299	1021 (29.6)	81.6	34.2
	≥300	742 (21.5)	63.3	33.9
**Patients and procedures**				
Sex	Male	794 (23.0)	105.1	56.5
	Female	2659 (77.0)	90.1	46.5
Age (years)	<40	794 (23.0)	99.8	53.5
	40–59	1572 (45.5)	92.1	47.7
	≥60	1088 (31.5)	91.3	46.3
Body mass index^b^	Underweight (≤18.5)	180 (5.2)	90.5	44.1
	Normal weight (>18.5 and ≤25)	1636 (47.4)	87.6	46.6
	Overweight (>25 and ≤30)	1034 (29.9)	97.3	50.3
	Moderated obesity (>30 and ≤35)	425 (12.3)	99.2	47.0
	Severe obesity (>35 and ≤40)	126 (3.7)	118.1	59.8
	Morbid obesity (>40)	53 (1.5)	112.4	50.9
Thyroid disease	Non-toxic solitary nodule	560 (16.2)	70.0	35.2
	Non-toxic multinodular goiter	1856 (53.7)	92.8	43.7
	Hyperthyroidism	337 (9.8)	97.5	45.9
	Graves' disease	323 (9.4)	106.1	47.4
	Malignant neoplasm	378 (10.9)	118.3	71.9
Bilateral procedure	Yes	2693 (78.0)	100.3	50.0
	No	761 (22.0)	69.9	35.2
Lymph node dissection	Yes	342 (9.9)	131.7	72.9
	No	2985 (86.4)	89.5	43.7
Supervision by more experienced surgeon	Yes	281 (8.1)	121.3	51.1
	No	3173 (91.9)	91.1	47.8
***Intraoperative variables***				
Difficulties locating parathyroid gland	Yes	636 (18.4)	115.2	56.6
	No	2818 (81.6)	88.7	45.4
Difficulties locating at least one recurrent nerve	Yes	820 (23.7)	100.8	55.3
	No	2634 (76.3)	91.4	46.3
Large goiter	Yes	603 (17.5)	111.5	46.3
	No	2851 (82.5)	89.8	48.4
Diving goiter	Yes	338 (9.8)	116.9	51.1
	No	3116 (90.2)	91.1	47.8
Hemorrhagic goiter	Yes	354 (10.2)	110.6	49.8
	No	3100 (89.8)	91.6	48.3
Fibrosis	Yes	291 (8.4)	101.5	55.4
	No	3163 (91.6)	92.9	48.0
Thyroiditis	Yes	178 (5.2)	113.8	47.2
	No	3276 (94.8)	92.5	48.6
Invasive cancer	Yes	35 (1.0)	155.8	72.2
	No	3419 (99.0)	93.0	48.1
Weight of thyroid specimen (g)	<20	1115 (32.3)	81.3	45.8
	20–39	1106 (32.0)	89.3	44.2
	40–59	512 (14.8)	103.2	51.2
	≥60	721 (20.9)	112.3	51.2

^a^ P-values adjusted by the False Discovery Rate method (to adjust for multiple comparisons): *P*<0.001 for all variables except fibrosis (*P* = 0.041) and age (*P* = 0.005)

^b^ Calculated as weight (kg) divided by height (m^2^).

^c^ Calculated as the number of years spent in practice since graduation.

The mean operative time varied from one center to another, ranging from 61 to 140 minutes. Operative time seemed to decrease with the accumulation of an individual surgeon’s experience, as well as with the volume of thyroidectomy performed over the year and the number of surgical procedures performed by the surgeon on the same day. At the patient level, mean operative time was shorter among women, older adults, and those with lower body mass index. Conversely, it was longer in cases of bilateral procedures, lymph node dissection, supervision by a more experienced surgeon, weighty thyroid specimen, and when difficulties had occurred during surgery.

Table 2 shows multivariate analyses of variables independently associated with operative time.

**Table 2:** Factors independently associated with operative time.

	Operative time (min)			
	Estimation	CI95%		*P* value
Intercept	86.0	84.8	87.2	<0.001
***Preoperative variables***				
**Surgeon**				
Length of experience (Ref <2 years)				
2–4	-13.2	-32.3	5.9	0.17
5–19	-21.8	-41.1	-2.4	0.02
≥20	-11.8	-32.9	9.3	0.28
No. of thyroidectomies performed per year (Ref <100)				
100–199	0.1	-14.9	15.1	0.99
200–299	-8.2	-27.9	11.5	0.43
≥300	-28.8	-50.0	-7.6	0.03
No. of thyroidectomies performed on same day (Ref = 1)				
2	-3.5	-6.2	-0.9	0.005
3	-7.7	-10.9	-4.4	<0.001
≥4	-11.7	-16.1	-7.4	<0.001
**Patients and procedures**				
Female sex	-7.7	-10.8	-4.5	<0.001
Age (by 10-year increase)	-1.7	-2.5	-0.9	<0.001
Body mass index (by 5-point increase)	3.2	2.0	4.3	<0.001
Non-toxic multinodular goiter	5.4	1.7	9.1	0.001
Hyperthyroidism	6.5	1.8	11.1	0.002
Graves' disease	9.9	4.5	15.4	<0.001
Malignant neoplasm	14.2	7.6	20.8	<0.001
Bilateral procedure	21.8	14.8	28.8	<0.001
Lymph node dissection	29.1	19.2	39.0	<0.001
Supervision by more experienced surgeon	20.0	13.0	27.0	<0.001
***Intraoperative variables***				
Difficulties locating parathyroid gland	7.0	3.7	10.3	<0.001
Difficulties locating at least one recurrent nerve	8.2	4.8	11.5	<0.001
Large goiter	6.8	3.3	10.3	<0.001
Diving goiter	4.6	0.9	8.3	0.007
Hemorrhagic goiter	7.5	3.5	11.5	<0.001
Fibrosis	7.6	3.6	11.6	<0.001
Thyroiditis	7.3	2.6	12.0	0.001
Invasive cancer	27.1	13.0	41.1	<0.001
Weight of thyroid specimen (by 100-g increase)	1.0	0.6	1.4	<0.001

The reference operative time was 86 minutes [95%CI, 84.8–87.2]. Operative time was significantly lower among experienced surgeons having practiced from 5 to 19 years (-21.8 min, 95%CI -41.1 to -2.4) or having performed at least 300 thyroidectomies per year (-28.8 min, 95%CI -50.0 to -7.6). There was also an inverse relationship with the number of thyroidectomies performed by the surgeon on the same day (-11.7 min for >3 procedures, 95%CI -16.1 to -7.4). By contrast, operative time was greatly increased when performing a lymph node dissection (+29.1 min, 95%CI 19.2 to 39.0), a bilateral procedure (+21.8 min, 95%CI 14.8 to 28.8), or when supervision of the procedure by a more experienced surgeon was required (+20 min, 95%CI 13.0 to 27.0). Malignant neoplasm (+14.2 min, 95%CI 7.6 to 20.8) was also associated with increasing procedure time, as well as any intraoperative difficulty such as invasive cancer (+27.1 min, 95%CI 13.0 to 41.1).

**Fig 1:**
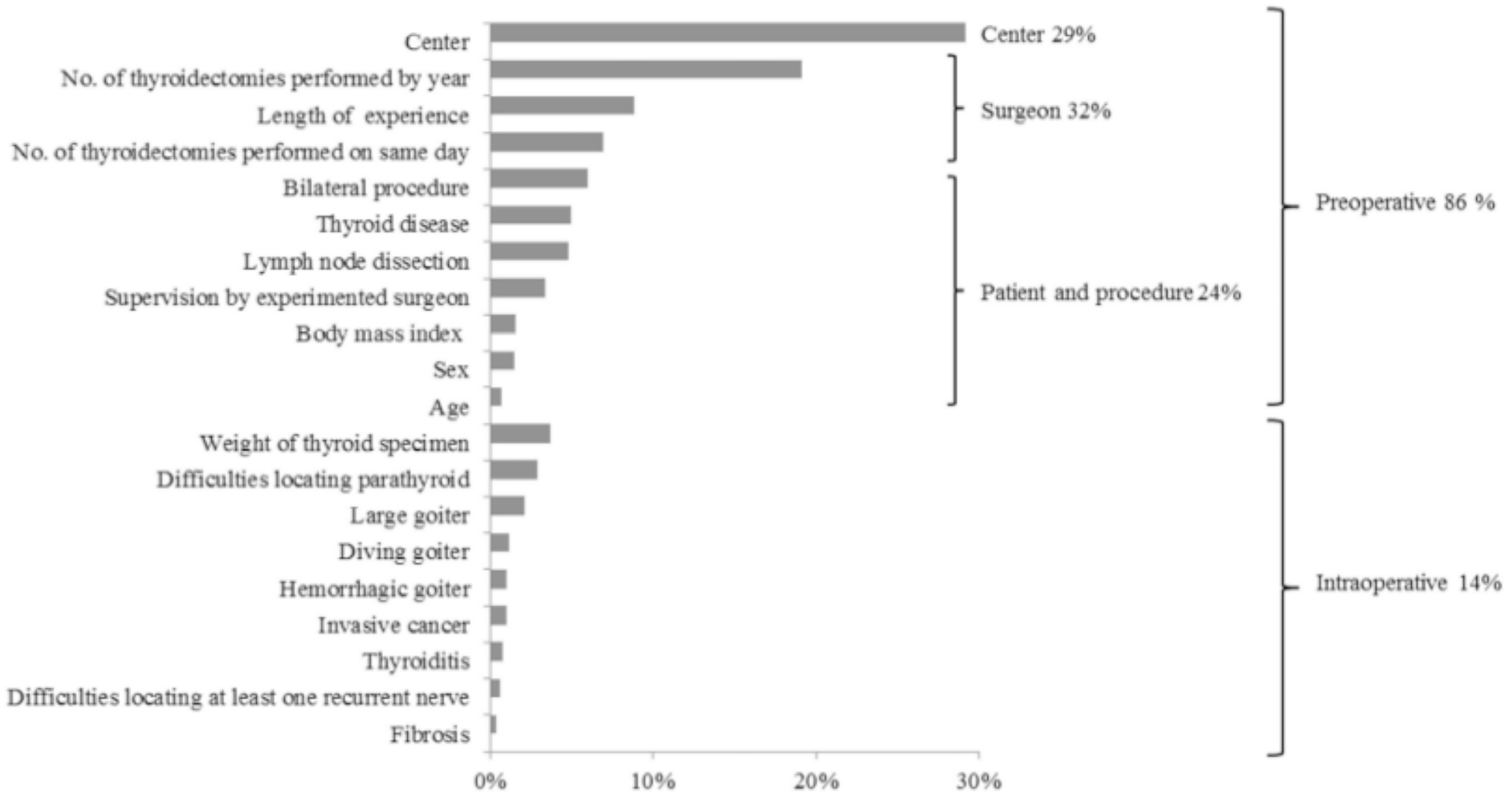
Relative importance of factors associated with operative time.

Overall, our multivariate model explained 63.4% of operative time variation, of which 85.9% [99%CI 82.6 to 88.7] was related to preoperative variables (Fig 1). Among these variables, surgeon characteristics accounted for 31.8% [99%CI 28.6 to 34.7] of variation (length of experience, 8.8% [99%CI 7.3 to 10.6]; volume of thyroidectomies per year, 19.1% [99%CI 16.5 to 21.4]; number of thyroidectomies on the same day, 6.9% [99%CI 5.3 to 8.9], center location for 29.1% [99%CI 25.4 to 32.6], and surgical procedure or patient for 23.7% [99%CI 20.3 to 27.4] (bilateral procedure, 6.0% [99%CI 4.2 to 7.7]; thyroid disease, 5.0% [99%CI 3.6 to 6.5]; lymph node dissection, 4.7% [99%CI 3.1 to 6.5]; supervision by a more experienced surgeon, 3.4% [99%CI 2.0 to 5.0]. The remaining 14.1% [99%CI 11.3 to 17.4] of variability accounted for in the multivariate model was attributable to intraoperative elements, ranging from 0.3% to 3.6% in accordance with unanticipated difficulties experienced during surgery (e.g. weight of thyroid specimen, 3.6% [99%CI 2.3 to 5.1]; or difficulties locating the parathyroid gland, 2.9% [99%CI 1.7 to 4.2]).

## Discussion

Based on the prospective collection of preoperative and intraoperative factors, this multicenter study provides tangible suggestions on operative time determinants. Our results highlight that observed variation in thyroidectomy duration can be largely explained by preoperative factors, and therefore be accurately anticipated. The major factors found to influence operative time included where the patient underwent the operation and by whom, rather than individual patient characteristics or the type of procedure planned. Furthermore, the occurrence of an unexpected event or technical difficulties at the time of the surgery had a negligible impact on its duration.

Previous models in general surgery studied lots of procedures and teams of surgeons and showed a negligible influence of surgeons and anesthesiologists on the overall time taken by team in operating room [11]. On the contrary, our study focused on a narrower population and recognizes the surgeon's individual experience as a key driver of performance focused on operative time from skin incision to closure. According to the “practice makes perfect” dogma, there is strong evidence that increasing surgical case volume and years of practice are associated with improved outcomes in a procedure-specific manner [12,13]. Following an initial learning curve, several studies noted a plateau phase, where increases in case volume or years of practice were no longer associated with improvements in surgical outcomes [14]. Based on the same dataset used in the present study, we have shown that the plateau for thyroidectomy spanned from 5 to 20 years of a surgeon's practice with regard to both postoperative recurrent laryngeal nerve palsy and hypoparathyroidism [7]. In a same fashion, the present study underlined that the mean operative time decreases when the surgeon’s length of experience increases, which supports the notion that a surgeon’s practice is both safer and faster once seniority is achieved. Those findings provide insights in the literature about determinants of operative time in thyroid surgery, which is scarce and traditionally focused on patients’ general and pathological characteristics [15].

Independently to operative time variations across hospitals and surgeons, several preoperative and intraoperative factors may contribute to the duration of thyroidectomy. In particular, we found that the number of thyroid procedures performed by a surgeon on the same day was a strong predictor of operative time. For a given surgeon, the busier the daily operation schedule, the more quickly operations were performed. A possible reason underlying this relationship may be that similar procedures are performed with a fixed operating room team. Previous studies have reported shorter operative times when repetitive procedures are performed by fixed teams [16]. Despite the pressure of a fuller schedule surgeons may experience a smoother workflow on these days. However, a major pitfall for experienced surgeons expecting to reduce operative time under a critical threshold would be to rush the surgery without complying with milestones for standardized procedures (e.g. not visualizing the recurrent nerve) [17]. The Holy Grail for achieving maximum health care value consists in finding the optimal balance between the quality and speed of a surgeon's performance, which involves meeting the highest standards while avoiding wasting staffing time in the operating room.

The strengths of our study include it’s a priori design to model the nature of the association between surgeons’ experience and performance in thyroid surgery. Because patient recruitment and data were recorded prospectively with great care, we avoided coding bias resulting from secondary utilization of administrative databases [18]. Based on the accuracy of the data, the patient features and other surgeon factors were partly controlled, and we considered the clustering of patients within surgeon and hospital center levels.

However, this work also has several limitations. First, the applicability of our results to other surgical fields is questionable, in view of the limited sample size of endocrine surgeons in academic referral centers. Thyroidectomy is considered a short procedure with quick access to the thyroid site and limited uncertainty in its duration. The highly standardizable steps in performing thyroidectomy allow the accurate anticipation of operative time due to a weak influence of intraoperative factors [6]. Longer procedures (i.e. oncologic abdominal surgery) are potentially less predictable [19] and require the consideration of variations in operative time related to intraoperative findings or emergency arrivals [20]. Second, despite adjusting simultaneously for procedure-specific, patient-specific, and surgeon-specific factors, the characteristics of thyroid diseases occasionally required surgeries in which complexity might not have been sufficiently captured. One-third of variation in operative time was not explained by our model, and we cannot exclude that other unknown or unmeasured factors might have explained part of this variation. In particular, the addition of intraoperative neuromonitoring [21] or ligasure [22], as well as checklist utilization, may impact thyroidectomy duration. Familiarity among the operating room staff [23] and a surgeon’s condition on a given day [24] are also essential for effective teamwork. Third, our work focused on surgical time from skin incision to wound closure. We did not consider the anesthesia induction time, which could represent up to one-third of total procedure time [25]. Assuming a fixed anesthesia induction time, we hypothesized that it was not highly subject to variability and would have a negligible influence on operating room schedules.

Operating room scheduling aims at planning where and when a procedure will takes place on a specific day [26]. Most hospitals use imperfect estimates as a reference to predict operative time based on the intuitive predictions of surgical staff or historical cases’ duration [27,28]. These data fail to ensure an acceptable level of planning quality [29]. Due to previous cases running late, scheduled procedures may be postponed and dropped from the schedule. As a consequence, patients may be inconvenienced by cancelation or delays in their surgeries [30].

By contrast, our study found explanatory variables of operative time that may optimize scheduling, based on concrete data considering the interplay of objective parameters. The statistical model explained a large proportion of variability in thyroidectomy duration based on preoperative factors. Hence, the development of a reliable prediction tool that accurately controls patient flow appears feasible for both working conditions and cost containment. Indeed, poor anticipation of surgical cases and non-compliance with the planned schedule can provoke workload disruption [31]. Beyond enhancing operating room productivity, the accuracy of the predefined operating room road map could maintain teamwork quality and patient safety [32].
